# Sonophoresis Using Ultrasound Contrast Agents: Dependence on Concentration

**DOI:** 10.1371/journal.pone.0157707

**Published:** 2016-06-20

**Authors:** Donghee Park, Gillsoo Song, Yongjun Jo, Jongho Won, Taeyoon Son, Ohrum Cha, Jinho Kim, Byungjo Jung, Hyunjin Park, Chul-Woo Kim, Jongbum Seo

**Affiliations:** 1 Department of Pathology, Tumor Immunity Medical Research Center, Cancer Research Institute, Seoul National University College of Medicine, Jongno-gu, Seoul, Republic of Korea; 2 Department of Biomedical Engineering, Yonsei University, Wonju, Gangwon-do, Republic of Korea; 3 Gumi Electronics & Information Technology Research Institute, Gumi, Gyeongsangbuk-do, Republic of Korea; 4 School of Electronic Electrical Engineering, Sungkyunkwan University, Suwon, Gyeonggi-do, Republic of Korea; Aristotle University of Thessaloniki, GREECE

## Abstract

Sonophoresis can increase skin permeability to various drugs in transdermal drug delivery. Cavitation is recognized as the predominant mechanism of sonophoresis. Recently, a new logical approach to enhance the efficiency of transdermal drug delivery was tried. It is to utilize the engineered microbubble and its resonant frequency for increase of cavitation activity. Actively-induced cavitation with low-intensity ultrasound (less than ~1 MPa) causes disordering of the lipid bilayers and the formation of aqueous channels by stable cavitation which indicates a continuous oscillation of bubbles. Furthermore, the mutual interactions of microbubble determined by concentration of added bubble are also thought to be an important factor for activity of stable cavitation, even in different characteristics of drug. In the present study, we addressed the dependence of ultrasound contrast agent concentration using two types of drug on the efficiency of transdermal drug delivery. Two types of experiment were designed to quantitatively evaluate the efficiency of transdermal drug delivery according to ultrasound contrast agent concentration. First, an experiment of optical clearing using a tissue optical clearing agent was designed to assess the efficiency of sonophoresis with ultrasound contrast agents. Second, a Franz diffusion cell with ferulic acid was used to quantitatively determine the amount of drug delivered to the skin sample by sonophoresis with ultrasound contrast agents. The maximum enhancement ratio of sonophoresis with a concentration of 1:1,000 was approximately 3.1 times greater than that in the ultrasound group without ultrasound contrast agent and approximately 7.5 times greater than that in the control group. These results support our hypothesis that sonophoresis becomes more effective in transdermal drug delivery due to the presence of engineered bubbles, and that the efficiency of transdermal drug delivery using sonophoresis with microbubbles depends on the concentration of microbubbles in case stable cavitation is predominant.

## Introduction

Sonophoresis, which uses ultrasound for transdermal drug delivery, is a non-invasive, painless method and is independent of electrical drug characteristics [1–3]. Sonophoresis can increase skin permeability to various drugs, including hydrophilic, lipophilic permeants, and large molecular weight compounds [4, 5]. Cavitation is generally recognized as the predominant mechanism of sonophoresis [6–8]. In TDD using sonophoresis, the nuclei of cavitation are mostly believed to be small bubbles trapped between the skin surface and transducer. Cavitation appears to cause disordering of the lipid bilayers and the formation of aqueous channels in the skin. Therefore, drugs can be deeply delivered into the skin [9]. The numerous studies suggested that sonophoresis does not induce any irreversible damage in the skin [10, 11]. Recovery of the skin barrier properties was indirectly evaluated by measurement of transdermal water flux and electrical resistance after ultrasound exposure. The result indicated that skin surface exposed to ultrasound eventually appears to recover to normal intact form within a day [12].

Cavitation is closely related to the frequency and pressure of ultrasound as well as the characteristics of bubbles [13, 14]. The likelihood of cavitation in natural skin conditions increases with lower frequency and higher negative pressure [6, 8]. Therefore, a relatively low frequency (< 200 kHz) is generally adopted to increase cavitation activity in sonophoresis [15, 16]. Low frequency ultrasound provides slowly fluctuating pressure field so that random size bubbles can be expanded to critical size and collapsed eventually [8, 17]. This violent event can create channels for drug delivery with strong influx streaming. Accordingly, researches in sonophoresis have been focused on transient cavitation which is related to rapid bubble collapse [6, 18]. On the other hand, when bubbles nearby skin are exposed to relatively low amplitude ultrasound, they will oscillate mildly, which is called stable cavitation, and cause microstreaming [9, 17, 19]. The microstreaming flow near skin induces flow fields that generate shear stresses, resulting in tension and stretching on skin surface that cause channels for TDD, allowing delivery of compounds [20].

In the previous works, we evaluated sonophoresis in the presence of engineered microbubbles which are widely used as ultrasound imaging contrast (UCA) agent [19, 21, 22]. UCAs have been also widely studied as cavitation seeds in drug delivery and have specific size distribution which can be related to narrow resonance frequency range. The expected phenomenon of bubble activity can be different according to the acoustic pressure field. Since the characteristics of presented microbubbles could be drastically changed after significant transient cavitations and microbubbles could not be replenished after initial application unlike sonoporation utilizing blood circulation, the pressure amplitude was limited to relatively low (~ 1 MPa) to predominantly utilize stable cavitation at the resonance frequency. Thru *in vitro* and *in vivo* experiments, sonophoresis utilizing stable cavitation of engineered microbubbles was confirmed to be more effective in the relatively high frequency range (e.g., >1 MHz).

In this presentation, we focused on UCA concentration in sonophoresis in the presence of UCA among many factors. In case of sonoporation, UCA are injected with pharmaceutics so that UCA concentration study has been limited due to safety of animals even though it is one of the most important parameters. In addition, the target location can be correlated with UCA concentration in sonoporation due to accumulated nonlinear effect during propagation path. On the other hand, sonophoresis provides a unique opportunity to study UCA concentration effect on drug delivery due to UCA applied on skin surface. The study was to prove that the efficiency of sonophoresis with microbubbles depends on the concentration of microbubbles. We supposed that TDD efficiency may be gradually increased until specific concentration of added microbubble and then dropped back above proper concentration. In order to obtain optimal range of UCA in sonophoresis, two different commercial UCAs and target molecules were examined. Ultrasound frequencies in the MHz range of 1.12 MHz to 2.47 MHz were adopted because commercial UCAs have a resonant frequency in the 1–5 MHz range. Two types of target molecules, glycerol and ferulic acid which have different viscosity, were used to confirm whether the efficiency of sonophoresis with microbubble shows similar effect on different situation if stable cavitation is predominant mechanism. Two analysis methods were used to quantitatively assess the efficiency of sonophoresis at each dose of UCA. First, optical clearing agent (OCA) delivery into porcine skin was conducted. OCAs such as glycerol, glucose, PPG/PEG, dimethyl sulfoxide (DMSO), and oleic acid cause dehydration of tissue ingredients and partial replacement of interstitial fluid [23, 24]. The efficacy of optical tissue clearing increase caused by sonophoresis with microbubbles was quantitatively evaluated using an optical property measurement system. Second, quantitative measures of porcine skin permeability using the Franz diffusion cell (FDC) were conducted. The target molecule delivered from the donor chamber to receptors through the skin is periodically withdrawn from the extraction port and then quantitatively analyzed by high performance liquid chromatography (HPLC).

## Materials and Methods

### Skin Preparation

Porcine skin samples were used for the experiments since the permeability of porcine skin is similar to that of human skin and due to its availability [25]. The skin samples were obtained from a local butcher (Parkdaljae LPC Co., LTD, Wonju, Kangwon, Korea), stored in a refrigerator at -19°C, and used for experiments within five days. They were retrieved and set for 30 minutes at room temperature before the process. Before beginning the experiment, the skin temperature reached 20–24°C, and the outer layer of skin was harvested with a microtome (Skin Grafting system-Zimmer, USA) at a thickness of 2.1–2.3 mm. The integrity of the skin sample was determined by measurement of the electrical resistance across the skin membrane. Resistance across the membrane was measured by passage of an electrical current across the skin membrane using two Ag/AgCl electrodes (MyoTrace, Hurev, Korea) at a 10 Hz frequency. The electrical resistance of skin used in experiments was in the range of 800–900 Ω with an average of approximately 850. Prepared skin was placed in a petri dish in phosphate buffer saline (PBS) solution for 20 minutes to hydrate the sample before the experiment, and the electrical resistance of the skin sample was measured.

### Target Molecules

Two target molecules, glycerol (Sigma–Aldrich srl, Milan, Italy) and ferulic acid (Sigma–Aldrich srl, Milan, Italy), were used according to the experimental analysis methods. First, glycerol was used in the optical clearing experiment since the efficacy of tissue optical clearing by sonophoresis with microbubbles can be quantitatively evaluated using the optical property measurement system according to each experimental condition. OCA has the potential to enhance light penetration through the skin due to its barrier activity against photon transmittance. Glycerol was diluted to a concentration of 70% with phosphate-buffered saline (PBS) based on previous studies [26, 27]. The PBS buffer used for dilution of glycerol and hydration of skin ranged in pH from 7.1–7.3. A total volume of 1 ml glycerol was applied on the porcine skin sample. Second, ferulic acid (molecular formula: C_10_H_10_O_4_, molecular Weight: 194.2, melting point: 174°C, and solubility: soluble in 95% ethanol (50mg/ml)), which has antioxidant activity, was used in the diffusion cell experiment. The efficiency of sonophoresis with microbubbles was analyzed for ferulic acid using HPLC. Five milliliters ferulic acid (10000 pm) were mixed in buffer solution (100 ml) with pH 7.

### Ultrasound contrast agents

Two commercial USAs, SonoVue^®^ (Bracco Diagnostics Inc., Milan, Italy) and Definity^®^ (Bristol-Myers Squibb Medical Imaging, Inc., USA), were used in both experiments. To determine the optimum concentration of UCA for TDD using sonophoresis, four different concentrations of UCA with target molecules were prepared. Each UCA was activated according to the manufacturer`s instructions. After activation, UCAs were mixed with glycerol at a volume ratio of 1:100, 1:1,000, 1:10,000, or 1:100,000 for the optical clearing experiment and with ferulic acid at a volume ratio of 1:100, 1:1,000, or 1:10,000 for the FDC experiment. The target molecule mixture was blended with Vialmix^®^ (Lantheus Medical Imaging, Inc., USA) for uniform distribution of UCAs. For example, a concentration of 1:1000 was made by blending target molecules of 999μl with UCA of 1μl using vial mixer for 45sec.

### Ultrasound

Two single-element transducers with a one-half-inch aperture were used in experiments at 1.12 MHz and 2.47 MHz, respectively. The impedance matching network was prepared to be tuned at 1.12 MHz and 2.47 MHz in the laboratory. To determine the ultrasound parameters, the acoustic pressure and field map were measured with a calibrated membrane hydrophone (UT1602-006, Precision Acoustics, UK). In the optical clearing experiment, an acoustic pressure of 1 MPa at a focus with a 1% duty cycle and 100 Hz pulse repetition frequency were used to generate a sonication intensity of 330 mW/cm^2^. In the diffusion cell experiment, an acoustic pressure of 1 MPa at a focus with a 10% duty cycle and 100 Hz pulse repetition frequency were used to generate a sonication intensity of 3.3 W/cm^2^. Even though two different intensity was used in the experiments, pressure amplitudes on skin surface were kept identical to avoid massive transient cavitation and to predominantly utilize stable cavitation.

### Light penetration by OCA

**Fig 1:**
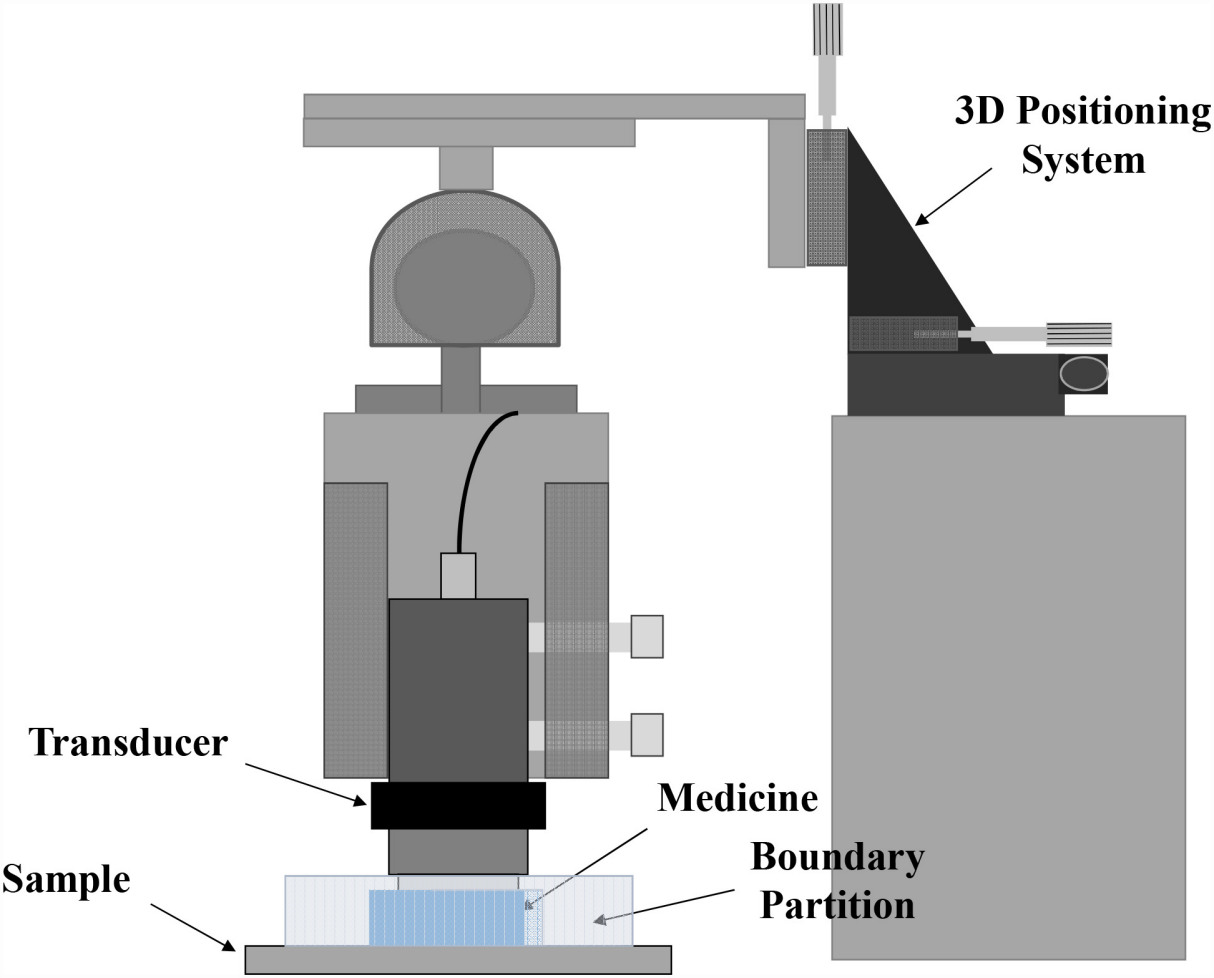
Experiment setup of ultrasound application.

The experimental setup for optical clearing of tissue is shown in Fig 1. A manual three-dimensional (3D) positioning system with an angular controller was used to control the position of the transducer on the skin sample. A flexible plastic ring (thickness, 15 mm; inner diameter, 21 mm) was used to prevent leakage of the glycerol mixture. Two types of transducers with a 12.5 mm diameter aperture at 1.12 MHz and 2.47 MHz were used in the experiment. After glycerol application, an acoustic intensity of 330 mW/cm^2^ was generated with an acoustic pressure of 1 MPa with a 1% duty cycle on the skin for 30 minutes in all sessions of ultrasound. Ultrasound transducers were positioned approximately 2 mm above the skin surface.

Three different experimental groups were formed to compare the efficiency of sonophoresis. The control group was designed to be a natural diffusion case without ultrasound sonication. The ultrasound group (1.12 MHz, 2.47 MHz) without UCA was designed to confirm the effect of general sonophoresis with high frequency (e.g., >1 MHz). In the ultrasound with UCA group, four concentrations of (1:100, 1:1,000, 1:10,000, 1:100,000) glycerol were used under two ultrasound frequencies. Each experimental condition was applied five times in order to allow for statistical analysis.

**Fig 2:**
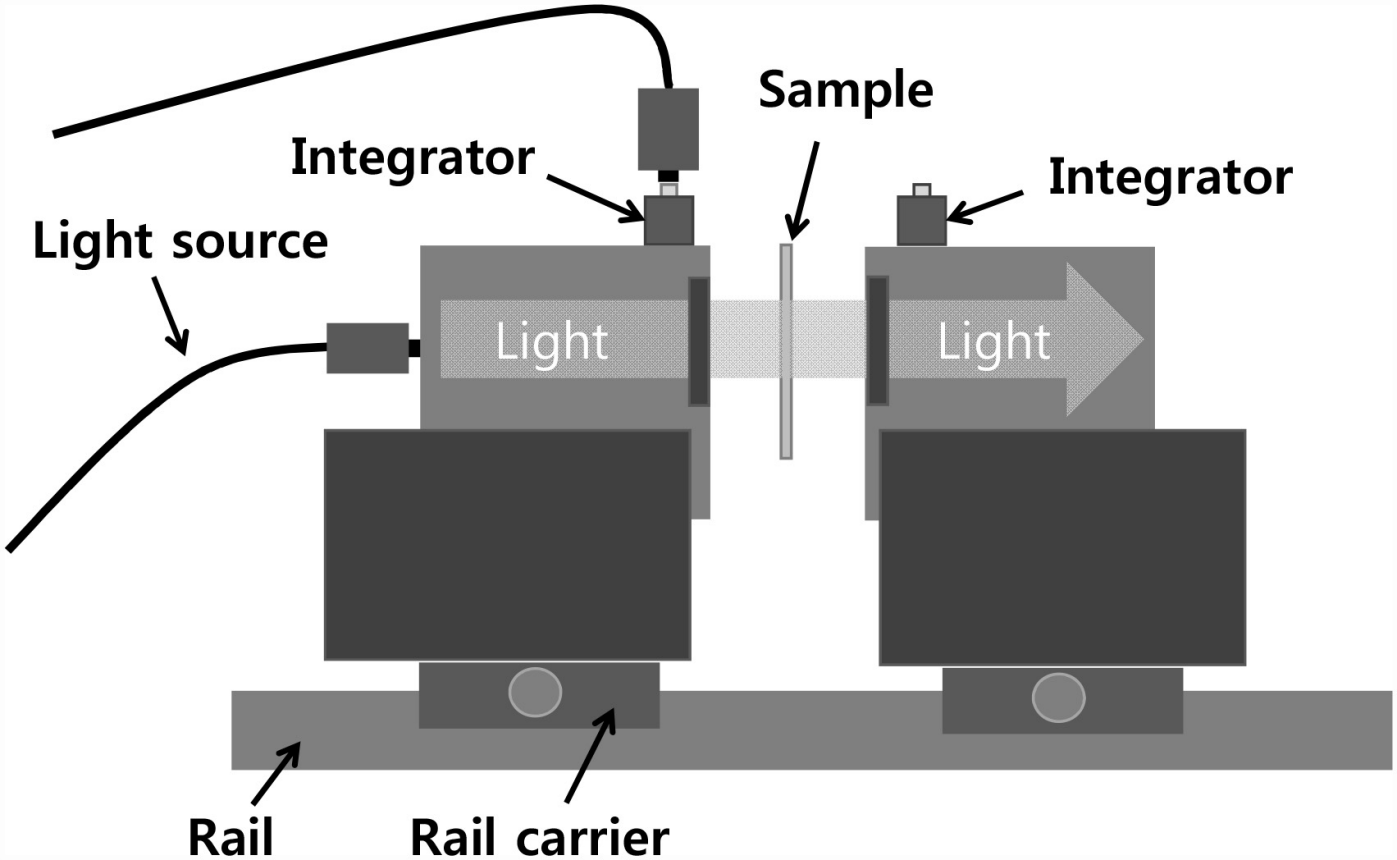
Optical properties measurement system.

To analyze the efficiency of sonophoresis, the optical measurement system was used as shown in Fig 2. Fig 2 illustrates the double-integrating-sphere system, which is widely used for measuring the optical properties of tissue. The double-integrating-sphere system consists of a quartz tungsten halogen lamp (66884, Oriel Instruments, USA), two integrating spheres (AvaSphere-30, Avantes, Netherlands), and two ports of an integrator. At the beginning of the experiment, the double-integrating-sphere system was calibrated without a sample using a reflectance material and an absorption material for reference value. The total reflection and transmission from the skin sample were measured by a VIS-NIR spectrometer (USB4000, Ocean Optics, USA) through the two ports of the integrator in the first and second integrating spheres, respectively. The inverse adding-doubling algorithm was used to determine the optical properties from these measurements. Light penetration was increased due to the reduced scattering coefficient resulting from glycerol. The efficiency of sonophoresis with UCA was evaluated based on the amount of light transmission.

### Franz diffusion cell experiment

An FDC with ferulic acid was used to estimate the efficiency of TDD using ultrasound with UCA. The 3D positioning system was used to control the angle and height of the ultrasound transducer. The temperature in a water tank was thermoregulated with a heating machine at 37°C. A magnetic stirrer was positioned under the Franz diffusion cell, and a magnetic stir bar was used to uniformly distribute the delivered ferulic acid in the receptor solution. The experimental groups were the same as those for the experiment of light penetration except for the case of 1:100,000, which was excluded based on the results of the optical clearing experiment. Each condition was tested five times to allow for statistical analysis of the data.

The excised and hydrated skin sample was mounted on an FDC where the stratum corneum side was facing the target molecules with/without transducer into the donor compartment, and the opposite side was facing the PBS solution into the receptor compartment. A total of 5 ml of ferulic acid solution mixed with each concentration of UCA was applied to the donor compartment, and then the parallel distance between the skin surface and ultrasound transducer was adjusted to 10 mm using the 3D positioning system to provide enough solutions. An acoustic intensity of 3.3 W/cm^2^ was generated with an acoustic pressure of 1 MPa with a 10% duty cycle on the skin for 30 minutes in all ultrasound sessions. A 1 mL solution in the receptor compartment was extracted to analyze the efficiency of sonophoresis at 10, 20, and 30 minutes after ultrasound sonication, and fresh PBS was supplied to the receptor at a volume equivalent to the extracted volume.

The composition of ferulic acid, which was delivered through the skin sample into the receptor compartment, was analyzed by HPLC (Agilent 1100 Series, Agilent, USA) equipped with a Zorbax Eclipse XDB-C18 (5 μm particle size, Agilent, USA) analytical column. The mobile phase was 0.1% phosphoric acid—acetonitrile (7:3), and the flow rate was set at 1 ml/min. A UV wavelength of 320 nm was used for detection of ferulic acid at a retention time of 10 minutes [28].

### Statistical analysis

Statistical analysis were performed using SPSS software (IBM SPSS Statistics, IBM Corporation, Chicago, IL). All data were calculated and presented as mean ± SD and p-value by t-test. The statistical differences between the natural diffusion and ultrasound groups were determined using a two-tailed, unpaired test. P-values 0.05 were regarded as statistically significant in all experimental groups.

## Results

### Light penetration by OCA

**Fig 3:**
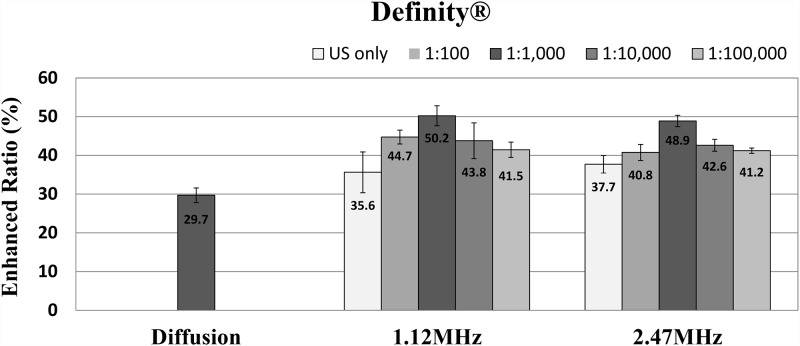
The efficiency of sonophoresis at two ultrasound frequencies for four different concentrations of Definity^®^.

**Fig 4:**
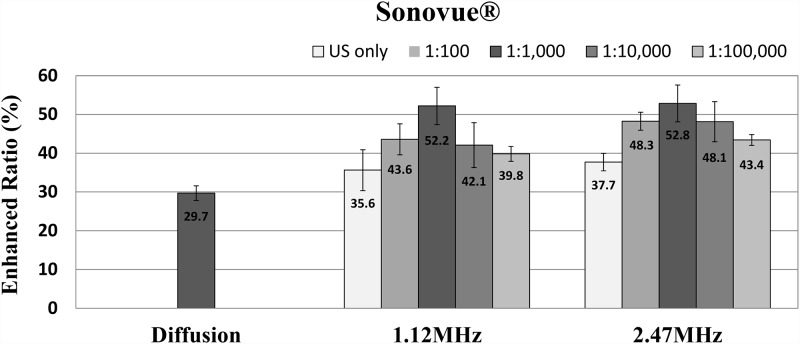
The efficiency of sonophoresis at two ultrasound frequencies for four different concentrations of SonoVue^®^.

When the OCA glycerol was delivered into the skin, the penetration of light increased due to a decrease in the scattering coefficient. The efficiency of TDD at two ultrasound frequencies for four concentrations of UCA is plotted in Figs 3 and 4. As seen in Fig 3, the natural diffusion without sonophoresis produced a 29.7% decrease in scattering coefficient. In all ultrasound sonication groups, the enhanced ratio of light penetration was greater than in the diffusion group, 35.6% at 1.12 MHz and 37.7% at 2.47 MHz without UCAs. The Definity^®^ with sonication at a frequency of 1.12 MHz improved the decrease in scattering coefficient by 44.7%, (P < 0.001) by t-test, 50.2% (P < 0.001), 43.8% (P < 0.005) and 41.5% (P < 0.001) at concentrations of 1:100, 1:1,000, 1:10,000, and 1:100,000, respectively. With Definity^®^ at a frequency of 2.47 MHz, the decrease in scattering coefficient was 40.8%, (P < 0.005) by t-test), 48.9% (P < 0.001), 42.6% (P < 0.005) and 41.2% (P < 0.001) at concentrations of 1:100, 1:1,000, 1:10,000, and 1:100,000, respectively. The statistically significant increment due to Definity^®^ was confirmed at all concentrations at both ultrasound frequencies.

The ultrasound sonication at 1.12 MHz and 2.47 MHz with Definity^®^ produced a larger decrease in scattering coefficient ratio compared to the natural diffusion case in all experimental groups. The results also show the concentration dependency of sonophoresis with UCA. The efficiency of TDD increased in accordance with the concentration increase of Definity^®^ to 1:1,000. However, the ratio at higher concentrations was reduced compared to that of the 1:1,000 concentration. This tendency according to the concentration of UCAs was similar for both frequencies. The TDD efficiency at a concentration of 1:1,000 showed the best results for both ultrasound frequencies. Although an ultrasound frequency of 1.12 MHz with Definity^®^ seemed to yield the best efficiency for glycerol delivery, the outcome differences between the two frequencies seem negligible.

Fig 4 shows the decrease in scattering coefficient by SonoVue^®^ at two ultrasound frequencies. The decrease was 43.6% (P < 0.005), 52.2% (P < 0.001), 42.1% (P < 0.05), and 39.8% (P < 0.005) at an ultrasound frequency of 1.12 MHz and 48.3% (P < 0.001), 52.8% (P < 0.001), 48.1% (P < 0.005), and 43.4% (P < 0.001) at an ultrasound frequency of 2.47 MHz at concentrations of 1:1,000, 1:10,000, and 1:100,000, respectively. The statistically significant effect on sonophoresis with SonoVue^®^ was also confirmed at all concentrations for both frequencies. The patterns of efficiency with respect to concentration of SonoVue^®^ were similar to that using Definity^®^. The ratios in all experimental groups with added UCA were higher than the control group without UCA. The light penetration caused by ultrasound with UCA increased at a lower concentration until a concentration of 1:1,000 but decreased at concentrations higher than 1:1,000. The ultrasound frequency of 2.47 MHz with SonoVue^®^ at a concentration of 1:1,000 showed the best result among all conditions in an optical clearing experiment, but the overall differences in light scattering due to frequency seem negligible, at least for this frequency range.

### Franz diffusion cell experiment

**Fig 5:**
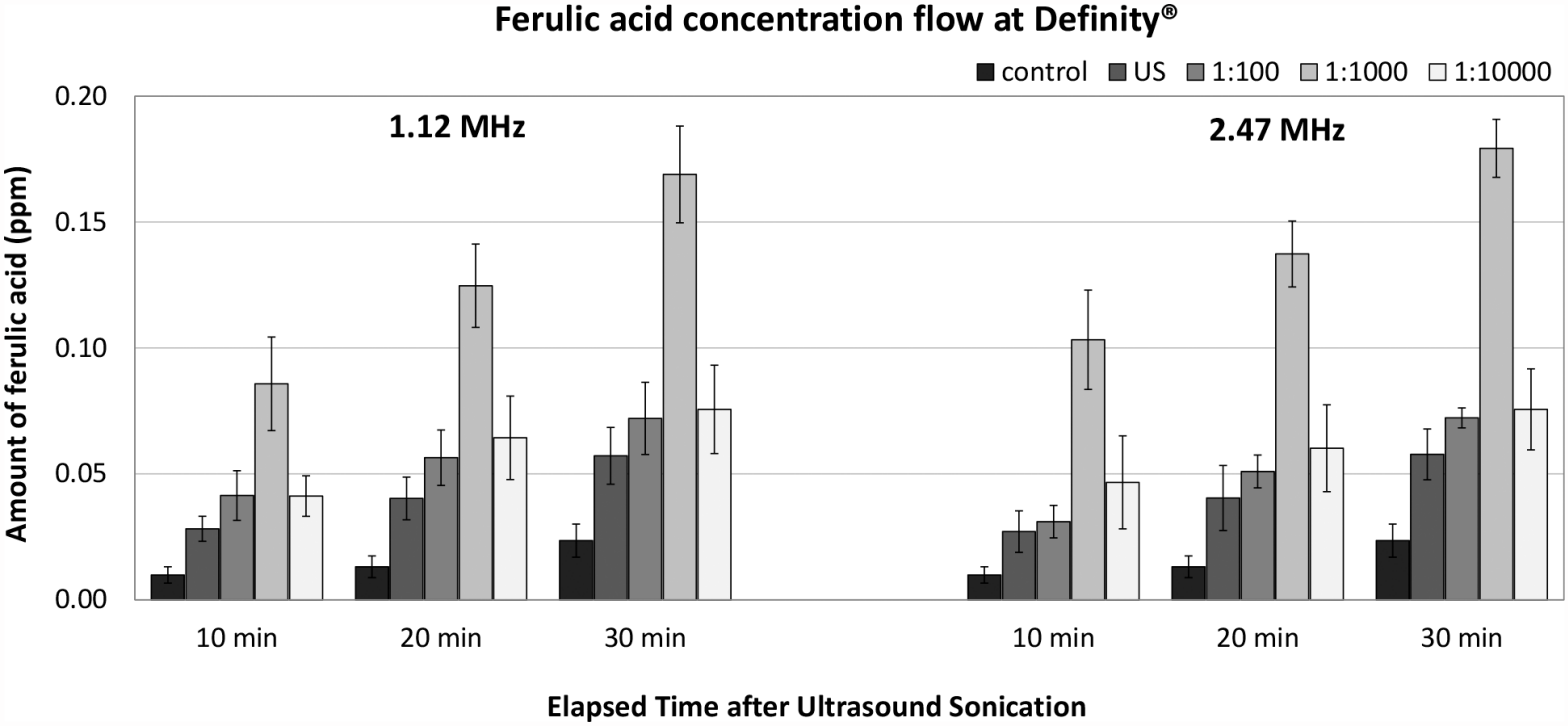
The efficiency of sonophoresis at two ultrasound frequencies and three different concentrations of Definity^®^.

**Fig 6:**
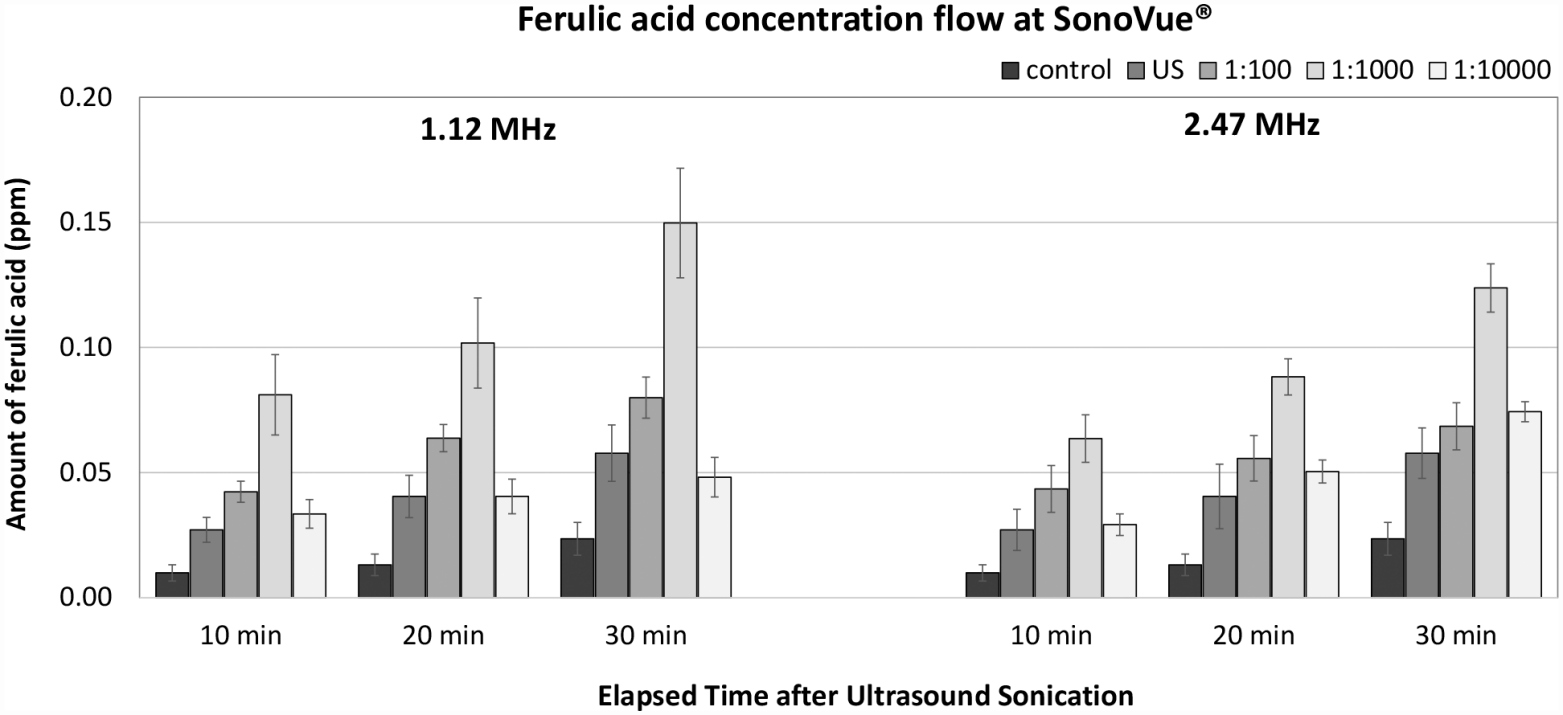
The efficiency of sonophoresis at two ultrasound frequencies for three different concentrations of SonoVue^®^.

The results for the efficiency of ultrasound with UCA are presented as parts per million (ppm) of ferulic acid, which is delivered to the receptor compartment through the skin by ultrasound with UCA. The composition of ferulic acid was measured by HPLC. The efficiency at two ultrasound frequencies with UCA is presented in Figs 5 and 6 according to the time interval after sonication.

The ferulic acid delivered by natural diffusion without ultrasound sonication was measured at 0.010, 0.013, and 0.024 at time intervals of 10, 20, and 30 minutes, respectively. The sonophoresis efficiencies with Definity^®^ were clearly increased compared to that of the diffusion group and ultrasound control group. The amount of ferulic acid delivered for sonophoresis with Definity^®^ was also gradually increased over a 30 minute interval. In all the sonophoresis with UCAs groups, the delivered amount of ferulic acid was at least 2.5 times (P < 0.05) larger than that of the diffusion group. The delivered amount of ferulic acid was the highest at concentrations of 1:1,000 in both frequencies. In the final time interval, the enhancement by sonophoresis at 1.12 MHz with a concentration of 1:1,000 was approximately 3.0 times (P < 0.001) greater than that in the ultrasound group without Definity^®^ and about 7.0 times (P < 0.001) greater than that in the ultrasound control group. Similarly, the delivered amount of ferulic acid at 2.47 MHz with a concentration of 1:1,000 was also 3.1 times (P < 0.001) greater than that in the ultrasound group without Definity^®^ and about 7.5 times (P < 0.001) greater than that in the ultrasound control group. There were no significant differences in TDD efficiency between frequencies of 1.12 MHz and 2.47 MHz in any concentration of Definity^®^. The results can be translated to average flux. As can be seen in Table 1, the average flux was 7.12 μg/cm^2^/h of diffusion over 30 minutes period, while sonophoresis with/without Definity^®^ increased the average flux at least more than twice. When concentration of 1:1,000 was used, the average flux reached highest values in both 1.12 MHz and 2.47 MHz (P<0.001).

**Table 1:** Steady state flux values of diffusion and ultrasound with Definity^®^ by Franz diffusion cell (mean ± S.D).

Definity^®^			
Concentration	Diffusion	1.12MHz	2.47MHz
	Flux (μg/*cm*^2^/*h*)	Flux (μg/*cm*^2^/*h*)	Flux (μg/*cm*^2^/*h*)
**0**	7.12±1.78	17.33±3.05	17.50±2.73
**1:100**	-	21.83±3.88	21.89±1.07
**1:1,000**	-	51.78±5.21	54.31±3.12
**1:10,000**	-	22.91±4.75	22.91±4.36

As seen in Fig 6, the sonophoresis efficiency with SonoVue^®^ had similar results to Definity^®^. In both UCAs, the amount of delivered ferulic acid was largest at a concentration of 1:1,000 at both 1.12 MHz and 2.47 MHz. The most significant difference between the two UCAs is that SonoVue^®^ appeared slightly more dependent on frequency that Definity^®^ in the given frequency range. The overall performance of sonophoresis with SonoVue^®^ at 2.47 MHz was visibly lower than at 1.12 MHz. The enhancement by sonophoresis at 1.12 MHz with a concentration of 1:1,000 was about 2.6 times (P < 0.001) greater than that in the ultrasound group without SonoVue^®^ and about 6.3 times (P < 0.001) greater than that in the ultrasound control group. Similarly, the delivered amount of ferulic acid at 2.47 MHz with a concentration of 1:1,000 was 2.1 times (P < 0.001) greater than that in the ultrasound group without SonoVue^®^ and about 5.2 times (P < 0.001) greater than that in the ultrasound control group. The significant effect of sonophoresis with SonoVue^®^ was confirmed at all concentrations for both frequencies. The absolute delivered amount of ferulic acid was transformed to average flux data as can be seen in Table 2. The relationship between average flux and SonoVue^®^ concentration follows then identical trends shown in Fig 6.

**Table 2:** Steady state flux values of diffusion and ultrasound with Sonovue^®^ by Franz diffusion cell (mean ± S.D).

Sonovue^®^			
Concentration	Diffusion	1.12MHz	2.47MHz
	Flux (μg/*cm*^2^/*h*)	Flux (μg/*cm*^2^/*h*)	Flux (μg/*cm*^2^/*h*)
**0**	7.12±1.78	17.33±3.05	17.50±2.73
**1:100**	-	24.22±2.23	20.75±2.55
**1:1,000**	-	45.37±5.94	37.50±2.62
**1:10,000**	-	14.59±2.14	22.51±1.09

## Discussion

Various factors, including ultrasound intensity, frequency, UCA concentration, distance between the skin surface and ultrasound transducer, and viscosity of target molecule solution, are thought to play important roles in sonophoresis with UCA for TDD. Among these factors, the TDD efficiency of sonophoresis using UCA was evaluated in this study with respect to the concentration of UCA. An optical clearing experiment was performed in advance of the FDC experiment. As a result, the efficiency of TDD increased in accordance with concentration increases of UCAs until 1:1,000. However, when a higher concentration of UCA was applied, the enhancement was reduced. TDD efficiency was continually increased until specific concentration of added microbubble and then dropped back above proper concentration. The TDD efficiency at a concentration of 1:1,000 in two types of UCA showed the best results at both frequencies, and the efficiency tendency according to the concentration of UCA was similar in both UCAs and at the two applied frequencies. Based on the results of the optical clearing experiment, the concentration of 1:100,000, which showed the lowest TDD efficiency, was excluded in the FDC experiment. The concentration of 1:1,000 showed the best TDD efficiency at both frequencies in the FDC experiment. Additionally, the FDC experiment resulted in an almost linear increase of delivered target molecule as the sonication duration increased. For example, the increase in delivered ferulic acid compared to that of the diffusion group was around 7–10 times in each 10-minute interval at a 1:1,000 concentration of Definity^®^. This indicates that the activity of microbubbles may be maintained relatively constantly over 30 minutes, even though ultrasound imaging of UCA shows a rapid reduction in brightness if UCA was applied by bolus injection [29].

As mentioned above, cavitation is generally considered the main mechanism of sonophoresis. In other words, an increase in cavitation activity can lead to enhancement of sonophoresis for TDD. We supposed that the low acoustic intensity applied in the previous experiment caused stable cavitation [22, 30], which seems to be a main mechanism independently of characteristics of drug such as viscosity [31]. A stable cavitation corresponds to a continuous oscillation of bubbles about the equilibrium radius in response to lower acoustic pressures in an acoustic field. The oscillation of bubbles around skin surface by stable cavitation leads to microstreaming, which is the unidirectional flow of fluid response to bubble dynamics in an acoustic field. The microstreaming flow may generate flow fields that develop shear stresses over a skin surface, resulting in tension and stretching on the skin surface to cause channel activation allowing drug delivery [2].

According to the UCA descriptions, the mean diameter of SonoVue^®^ is 2.5 μm, with 90% of the bubbles having a diameter less than 8 μm and 99% having a diameter less than 11 μm. The mean diameter range of Definity bubbles is 1.1 μm ~ 3.3 μm, with 98% having a diameter less than 10 μm. In summary, both UCAs used in experiments have a roughly similar size distribution. The resonance frequency range of each UCA was practically measured at about 1 ~ 3 MHz [32] for Definity^®^ and 1.6 ~ 3.1 MHz [33] for SonoVue^®^. In all experiments using Definity^®^, the differences in TDD efficiency according to ultrasound frequency were slight. Similarly, the difference in TDD efficiency was also not significant for the two ultrasound frequencies in experiments using SonoVue^®^. The similarity may be due to the size and resonance frequency range of microbubbles for both UCAs.

Although both UCAs had a roughly similar size distribution, they had several differences, including the type of shell material and the inner gas and bubble concentrations per ml, as well as shell stiffness and friction. In the case of Definity^®^, the perflutren lipid microspheres are composed of octafluoropropane encapsulated in an outer lipid shell (DPPA, DPPC, and DPPE), and each mL of the milky white suspension contains a maximum of 1.2 X 10^10^ perflutren lipid microspheres [34]. A SonoVue^®^ system is made up of a combination of phospholipids and pharmaceutical grade polyethyleneglycol, and the gas phase is sulfur hexafluoride (SF_6_). The maximum bubble concentration of SonoVue^®^ is 5 X 10^8^/ml. There is a 24-fold difference in bubble concentration between Definity^®^ and SonoVue^®^. In both experiments, we added UCAs as a volume ratio, which was not considered for bubble concentration in the UCA description. As a result, the TDD efficiency was correlated with the added volume ratio for both UCAs. These results indicate that each commercial UCA has a different efficiency for the same ultrasound conditions according to the character of the UCA. In other words, the applied ultrasound pressure to both UCAs was identical, but the activity of microbubbles may be different due to the character of the UCA, including shell stiffness, shell friction, and types of shell material and inner gas [35–37]. Therefore, the cavitation effect varies based on microbubble activity, and further studies are required to clarify the individual type of bubble activity.

Considering the target molecules used in the presentation were relatively small (< 1 kDa), the current result may not provide enough information for larger drug molecules. However, numerous studies have shown usefulness of sonophoresis on delivery of various drug molecules such as fentanyl, caffeine, heparin, ketoprofen and insulin [19, 38–40]. Among these therapeutic drugs, we might use sonophorosis with UCA for the delivery of insulin whose molecular weight is approximately 6 kDa. In fact, noninvasive transdermal delivery of insulin has received great attention due to diabetes treatment, one of the most costly diseases in all patient populations and age groups [41, 42]. Non-invasive insulin delivery through the skin is a preferable candidate for diabetic treatment instead of traditional invasive and painful subcutaneous insulin injections and improvement of sonophoresis will provide great opportunity.

## Conclusion

The dependence on UCA concentration for sonophoresis was evaluated though two types of experiments. Our results from these experiments indicated that the concentration of UCA significantly influences the efficiency of sonophoresis with UCA. Specifically, a concentration of 1:1,000 with two types of UCA yielded the best result for TDD efficiency among the several concentrations used in the two experiments. These results support our hypothesis that sonophoresis becomes more effective due to the presence of engineered bubbles such as those of UCA, and the efficiency of sonophoresis with microbubbles depends on the concentration of microbubbles. Further studies will focus on: (i) the manufacturing of bubbles encapsulated with a target molecules for TDD; (ii) an efficiency comparison of sonophoresis using manufactured bubbles and commercial UCAs for TDD.
